# Predictors of Atrial Fibrillation in Patients with Embolic Stroke of Unknown Etiology and Implantable Loop Recorders—Further Insights of the TRACK AF Study on the Role of ECG and Echocardiography

**DOI:** 10.3390/jcm12206566

**Published:** 2023-10-17

**Authors:** Dennis Höwel, Patrick Leitz, Gerrit Frommeyer, Martin A. Ritter, Florian Reinke, Anna Füting, Nico Reinsch, Lars Eckardt, Simon Kochhäuser, Dirk G. Dechering

**Affiliations:** 1 Department of Cardiology, St. Marienhospital Vechta, Marienstr. 6-8, 49377 Vechta, Germany; 2 Department of Cardiology II—Electrophysiology, University Hospital Münster, 48149 Münster, Germany; 3 Department of Neurology, University Hospital Münster, 48149 Münster, Germany; 4 Department of Medicine, Witten/Herdecke University, 58455 Witten, Germany; 5 Department of Internal Medicine/Cardiology, Marienhospital Osnabrück, 49074 Osnabrück, Germany

**Keywords:** ESUS, atrial fibrillation, ECG, echocardiography, P-wave, premature atrial contraction

## Abstract

**Aims**—Electrocardiography (ECG) and echocardiographic left atrial (LA) parameters may be helpful to assess the risk of atrial fibrillation (AF) in embolic stroke of unknown etiology (ESUS) and could therefore guide intensity of ECG monitoring. **Methods**—1153 consecutive patients with ischemic stroke or transient ischemic attack (TIA) were analyzed. An internal loop recorder (ILR) was implanted in 104 consecutive patients with ESUS. Multiple morphologic P-wave parameters in baseline 12-channel ECG and echocardiographic LA parameters were measured and analyzed in patients with and without ILR-detected AF. Using logistic regression, we evaluated the predictive value of several ECG parameters and LA dimensions on the occurrence of AF. **Results**—In 20 of 104 (19%) patients, AF was diagnosed by ILR during a mean monitoring time of 575 (IQR 470–580) days. Patients with AF were significantly older (72 (67–75) vs. 60 (52–72) years; *p* = 0.001) and premature atrial contractions (PAC) were more frequently observed (40% vs. 2%; *p* < 0.001) during baseline ECG. All morphologic P-wave parameters did not show a significant difference between groups. There was a non-significant trend towards a larger LA volume index (31 (24–36) vs. 29 (25–37) mL/m^2^; *p* = 0.09) in AF patients. **Conclusions**—Age and PAC are independently associated with incident AF in ESUS and could be used as markers for selecting patients that may benefit from more extensive rhythm monitoring or ILR implantation. In our consecutive cohort of patients with ESUS, neither morphological P-wave parameters nor LA size were predictive of AF.

## 1. Introduction

Cardio embolism due to atrial fibrillation (AF) is responsible for some embolic strokes of unknown etiology (ESUS). Diagnosis of a- or oligosymptomatic AF is often difficult because of its paroxysmal occurrence and the intermittent character of ECG monitoring [1]. Implantable loop recorders (ILR) have significantly improved AF detection [2] but are an invasive diagnostic tool with an increased burden on health care expenditures.

Our study as well as others have shown an AF incidence of 18–30% in patients with cryptogenic strokes [3,4,5]. A meta-analysis of 50 studies comprising 11,658 post-stroke patients found an overall detection rate of AF of 23.7% [6]. In addition, the large randomized LOOP trial recently demonstrated AF in 32% of ILR patients as compared to 12% in the control group [7].

Direct oral anticoagulants have generally made oral anticoagulation easier and safer. However, recent large randomized trials have shown that there seems to be no benefit in anticoagulating every patient with ESUS [8,9]. Hence, there is still a strong need to identify patients that benefit from AF screening and oral anticoagulation due to AF.

AF leads to complex alterations in the atrial myocardium referred to as atrial remodeling. This cannot only be induced by AF itself but also by different other cardiac and extra-cardiac factors in the absence of AF, e.g., structural heart disease, (sub-clinical) heart failure, aging, cardiovascular risk factors, etc. Atrial remodeling is a crucial factor in AF initiation and perpetuation. Clinically, an increased left atrial (LA) size and widened or heightened P-wave in the ECG are signs of atrial remodeling and thus AF. Indeed, these parameters are consistently associated with a higher risk for AF in multiple clinical settings [10,11,12,13,14,15,16]. However, all of the studies investigating the impact of P-wave morphology and LA dimensions were limited by their intermittent ECG diagnostics of AF, which mostly consisted of only 24 h of monitoring during follow-up. This methodological flaw leads to vast underdiagnosis of asymptomatic paroxysmal AF.

As of now, it is unknown if electrical (i.e., P-wave) or mechanical atrial parameters (i.e., LA size) are more closely linked to the occurrence of (asymptomatic) AF. We thus investigated different P-wave and LA parameters in our prospective TRACK-AF study with continuously monitored patients with cryptogenic stroke to identify possible predictors for the presence of AF in patients with ESUS [10,11,12,13,14]. Furthermore, we analyzed if electrical or mechanical factors of atrial remodeling better predict the presence of AF in continuously monitored patients.

## 2. Materials and Methods

### 2.1. Study Population

The study population consisted of consecutive patients from the TRACK-AF prospective study [17]. The study protocol was approved by the local ethics committee and registered at ClinicalTrials.gov (NCT02641678).

The eligibility criteria were cryptogenic stroke according to the Trial of Org 10,172 in Acute Stroke Treatment (TOAST 5b) [18]; exclusion criteria were incomplete workup and potential competing sources of embolism.

All patients underwent a thorough diagnostic workup protocol before inclusion. The patients’ medical history including typical cardiovascular risk factors and prior medication were recorded. All patients had a typical embolic stroke pattern on MRI or CT, one 12-lead ECG upon admission, 72 h continuous ECG monitoring (Dräger Infinity Delta, Lübeck, Germany) on our stroke unit, and 24 h Holter ECG (Spider View, Ela Medical, Sorin Group, Milan, Italy) within the first 5 days of admission. Only the baseline 12-lead ECG upon admission was taken into account for further analysis as mentioned below; Holter ECG and continuous monitoring were solely used to rule out manifest atrial fibrillation. Transesophageal/transthoracic echocardiography and ultrasound imaging of the brain supplying arteries were performed in all patients. Only patients without AF during the first ECG monitoring days were included and a previously validated implantable loop recorder (ILR) (Reveal XT, Medtronic, Minneapolis, MN, USA) was implanted [19]. The ILR is capable of detecting AF automatically and independently from heart rate or symptoms. Each recording of the ILR was reviewed by two experienced electrophysiologists and had to be independently classified as an AF episode lasting at least 30 s.

### 2.2. ECG Parameters

**Figure 1:**
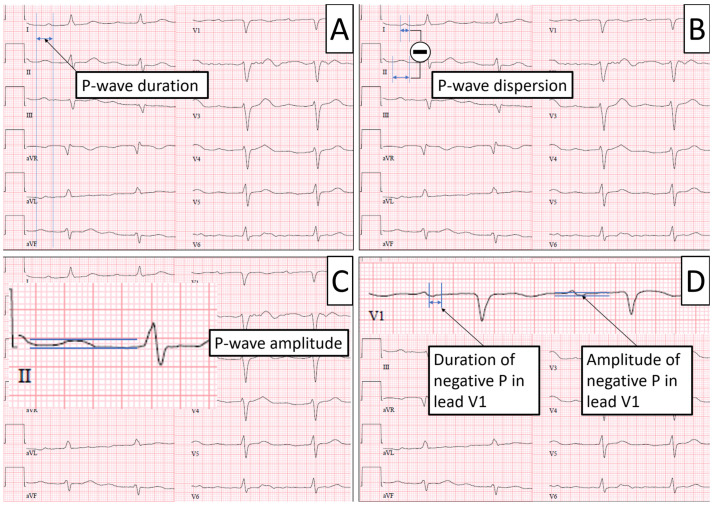
Measurement of ECG parameters. Panel (**A**)—P-wave duration is measured between the earliest detection of atrial depolarization in any lead (=P onset; left bar) to the latest detection of atrial depolarization (=P offset; right bar). Panel (**B**)—P-wave dispersion is defined as the difference between the longest and shortest P-wave duration when measured individually in each lead (in this case, longest in lead II and shortest in lead I). Panel (**C**)—P-wave amplitude was defined as the maximum amplitude measured in any lead from baseline to positive peak of P-wave. Panel (**D**)—Typical biphasic P-wave in lead V1; duration (left) and amplitude (right) of the negative portion were measured as shown.

The baseline 12-lead ECG was registered upon admission and printed at a paper speed of 50 mm/s on recording paper. The tracings were evaluated independently by two physicians. P-wave duration was defined as the interval between the earliest detection of atrial depolarization in any lead and the latest detection of atrial depolarization in any lead [13]; a P-wave duration <110 ms is considered normal [11]. P-wave dispersion was defined as the difference between the longest and shortest P-wave duration when measured individually in each lead; the normal value for P-wave dispersion is <40 ms [11]. P-wave amplitude was measured from baseline to the positive peak of the P-wave in all limb and augmented limb leads [12]; the maximum obtained value was used for analysis. The P-wave in lead V1 is typically biphasic with a second negative portion of which duration and amplitude were measured [14]. In addition, lead II was checked for a biphasic P-wave [10]. Furthermore, the baseline ECG was screened for premature atrial contractions (PACs) [20,21]. Figure 1 shows an example of how the measurements were obtained.

### 2.3. Echocardiographic Parameters

Transthoracic and transesophageal echocardiographic images were acquired by experienced physicians according to an internal standard protocol; the images were digitally stored on a local server. The images were then reviewed independently by a different physician using a specialized image analysis software (Image Arena, Tomtec Corporation, Chicago, IL, USA). LA diameter in parasternal long-axis view (normal value < 40 mm) and estimated LA volume (area-/length method) in apical 2- and 4-chamber views were measured according to the recommendations of the American Society of Echocardiography and the European Association of Cardiovascular Imaging [22]. The estimated LA volume was adjusted for body surface area calculated by the Mosteller formula [23]. A left atrial volume index < 34 mL/m^2^ is considered normal.

### 2.4. Statistical Analysis

We used IBM SPSS Statistics 24.0 (IBM Corporation, Somers, NY, USA) for statistical analysis. Shapiro–Wilk and Kolmogorov–Smirnov tests were used to test normality. Mann–Whitney U-test and Fisher’s exact tests were used to compare metric and dichotomous variables, respectively.

We created several binary logistic regression models with the occurrence of AF as the dependent variable. In one model, we forced age, gender, and univariately associated variables (*p* < 0.15) into the model, while accounting for possible multicollinearity. As a maximum, we included 3 variables into logistic regression models and used the variance inflation factor to make spurious associations less probable. To test for robustness of the statistical models, we additionally forced single variables into the model that could be meaningful from a clinical perspective (e.g., diabetes mellitus, congestive heart failure, hypertension as risk markers for AF).

## 3. Results

### 3.1. Study Population

A total of 1153 consecutive patients (539 female, 47%) were admitted to our stroke unit with the diagnosis of ischemic stroke or transient ischemic attack (TIA) and screened for eligibility. 104 patients were consecutively enrolled and implanted with an ILR. AF was detected in 20 patients (19%) in a median follow-up time of 575 (IQR 470–580) days. Median age was 63 years (IQR 52–74 years); 43% were women. Details of the baseline assessments are shown in Table 1.

### 3.2. Univariate Analysis

#### 3.2.1. Baseline Parameters

Patients with AF were significantly older (72 (67–75) vs. 60 (52–72) years; AF vs. no AF; *p* = 0.001) and—because of the higher age—had a significantly higher CHA_2_DS_2_-Vasc Score (5 (4–6) vs. 4 (3–5) points; AF vs. no AF; *p* = 0.02). All other baseline parameters (gender, body mass index, hypertension, diabetes, chronic heart failure, vascular disease) did not differ between the two groups.

#### 3.2.2. Echocardiographic Parameters

Median LA diameter in parasternal long axis was 38 mm, median LA volume was 56 mL, and indexed LA volume (LAVI) was 29 mL/m^2^; none of these measures showed a significant difference between patients with detected AF and those without. A non-significant trend towards a larger LAVI in patients with AF was observed (31 vs. 29 mL/m^2^; *p* = 0.09). Detailed results of the univariate analysis are shown in Table 2.

#### 3.2.3. ECG Parameters

All obtained conduction parameters were within normal range. Patients with AF during follow-up showed a non-significant trend towards a shorter median -P-wave dispersion (10 ms vs. 20 ms; *p* = 0.08) and shorter duration of negative P-wave in V1 (60 ms vs. 67 ms; SD; *p* = 0.097). The incidence of premature atrial contractions (PACs) in the baseline ECG was significantly greater in the AF group when compared to the non-AF group (40% vs. 2%; *p* < 0.0001). Detailed results of the univariate analysis are shown in Table 2.

### 3.3. Multivariate Analysis

In the multivariate analysis, age (*p* = 0.01) and presence of PACs in the baseline ECG (*p* < 0.001) independently showed a strong positive association with the occurrence of AF in our study population. Neither echocardiographic left atrial parameters nor any other baseline or univariately associated ECG parameter showed a significant correlation in the multivariate analysis. Detailed results of the multivariate analysis are given in Table 3.

## 4. Discussion

Continuous ECG monitoring with ILRs poses the great opportunity of identifying patients that may benefit from an oral anticoagulation due to previously undetected AF. To increase the yield of AF detection and thus to avoid unnecessary ILR implantations, the selection of patients that particularly benefit from continuous ECG monitoring or a costly and invasive ILR implantation is of major clinical relevance.

In this study, we showed that next to age, only the presence of PACs in the baseline ECG predicted AF in a continuously monitored patient cohort with ESUS. Interestingly, although LA size is often used as a strong AF risk marker, the simple method of obtaining the presence of PACs from a standard 12-lead ECG predicted AF over and beyond highly standardized measurements of LA dimensions. Therefore, the results of this study could help to identify patients that should undergo prolonged monitoring or ILR implantation after ESUS. Older age and the presence of PACs in the baseline ECG might be useful as simple selection tools for extended rhythm monitoring.

To our knowledge, this is the first study that confirms the association of PACs and future AF in a standard resting ECG in the setting of ESUS. This result is in line with a previous analysis by our group [20] and Gladstone, et al. [21], who found a strong association of PACs detected by Holter monitoring in this specific patient collective. Age is a strong independent risk factor for the prevalence of AF in general, which has been shown in multiple previous studies [24]. It comes as no surprise that this association was also detectable in our patients with ESUS.

In the context of AF-predicting P-wave parameters, Dilaveris, et al. [11] were able to show that patients with previously diagnosed paroxysmal AF showed a significantly longer P-wave dispersion in comparison to a control group. Compared to our results, we can speculate that they could be due to the fact that we studied patients with no history of AF, so that our collective presumably had a lower AF burden before enrollment and thus less marked atrial remodeling, leading to an increase in P-wave dispersion. A large primary care population-based study by Nielsen et al. [13] analyzed a total of >280,000 ECGs and found an increased risk of incident AF for long P-wave duration (≥120 ms) which we could also not replicate in our cohort of patients with ESUS.

The strength of our study is the validated continuous ECG monitoring of all included patients, so that the rate of undetected AF is negligible. Also, all patients underwent a very thorough workup, especially a very long ECG monitoring before inclusion, so that only patients with true ESUS were included. This combination of very selective inclusion and continuous monitoring by means of ILR is not found in previous similar studies.

This study uses atrial fibrillation as an endpoint. To this point, it is still unclear which therapeutic consequences arise from an AF diagnosis derived from extensive screening. The LOOP trial [7] has not found a clinical benefit of oral anticoagulation concerning stroke risk in patients with stroke risk factors and AF episodes of > 6 min detected by an implanted loop recorder. In line with these results, the recently published NOAH-AFNET6 trial [25] did also not show a benefit of oral anticoagulation use in patients with atrial high-rate episodes detected by implanted pacemaker or defibrillator devices, concerning a combined endpoint of cardiovascular death, stroke or systemic embolism. On the other hand, there is clear evidence for drastically reducing stroke risk with oral anticoagulation in the setting of stroke and clinical atrial fibrillation [26]. Future trials addressing the question of which combination of AF burden and risk factors in screening-detected AF should lead to establishing an oral anticoagulation in an individual patient are necessary.

### Limitations

First, we have a relatively small sample size for analysis. This is a possible reason why we could not show an association of LA-size and P-wave metrics in our patient group. However, our presented results were robust in subgroups and with different statistical models. A second limitation might be that all ECG measurements were obtained by manually measuring paper-based ECGs by two experienced electrophysiologists in contrast to an automated or software-supported analysis of digital ECGs, which could potentially be more accurate. Even if more precise, such a software would be more difficult to implement on a large scale. However, we tried to find a simple clinically relevant tool to select patients who would benefit most from ILR implantation.

## 5. Conclusions

Age and premature atrial contractions in a baseline ECG are independently associated with the diagnosis of atrial fibrillation in patients with embolic stroke of unknown etiology in a patient population without previously known atrial fibrillation. The very easily obtainable 12-lead ECG measurement may aid to select patients who benefit most from intensive rhythm monitoring or implantation of a loop recorder after ESUS.

## Figures and Tables

**Table 1:** Baseline Characteristics.

Characteristics	All (*n* = 104)	Atrial Fibrillation (*n* = 20)	No Atrial Fibrillation (*n* = 84)	*p*-Value
Age, years, median (IQR)	63 (52–74)	72 (67–75)	60 (52–72)	0.001
Female, n (%)	45 (43.3)	10 (53)	35 (41)	0.62
Body mass index, kg/m^2^, median (IQR)	27 (24–29)	26 (24–29)	27 (24–29)	0.89
CHA_2_DS_2_-Vasc Score (IQR)	4 (3–6)	5 (4–6)	4 (3–5)	0.02
Arterial hypertension, n (%)	77 (74)	16 (84)	61 (72)	0.39
Diabetes, n (%)	24 (23)	4 (21)	20 (24)	0.82
Congestive heart failure, n (%)	4 (4)	2 (11)	2 (2)	0.15
Vascular disease, n (%)	17 (16)	3 (16)	14 (17)	0.94

Abbreviations: IQR = interquartile range; n = number.

**Table 2:** Univariate Analysis.

Characteristics	All (*n* = 104)	Atrial Fibrillation (*n* = 20)	No Atrial Fibrillation (*n* = 84)	*p*-Value
ECG parameters				
P-Wave duration, ms, median (IQR)	110 (100–120)	110 (100–120)	110 (100–120)	0.68
P-Wave dispersion, ms, median (IQR)	20 (10–30)	10 (10–20)	20 (10–30)	0.08
P-Wave amplitude, mV, median (IQR)	0.1 (0.08–0.12)	0.1 (0.08–0.12)	0.08 (0.08–0.12)	0.76
Dur. neg. P in V1, ms, median (IQR)	60 (60–80)	60 (50–70)	60 (60–80)	0.10
Amp. neg. P in V1, mV, median (IQR)	0.1 (0.05–0.1)	0.1 (0.05–0.1)	0.1 (0.05–0.1)	0.42
P wave biphasic in II, n (%)	11 (10)	2 (13)	9 (10.1)	0.97
Premature atrial contractions present, n (%)	10 (10)	8 (40)	12 (2)	<0.001
Echocardiographic parameters				
LA diameter plax, mm, median (IQR)	38 (35–41)	39 (36–42)	38 (35–41)	0.15
LA area ap4, cm^2^, median (IQR)	18 (17–22)	18 (16–24)	18 (17–22)	0.33
LA area ap2, cm^2^, median (IQR)	19 (16–22)	20 (17–23)	19 (16–22)	0.17
LA length, mm, median (IQR)	52 (48–55)	52 (47–57)	52 (48–55)	0.45
LA volume, mL, median (IQR)	56 (48–75)	58 (49–80)	56 (47–75)	0.20
LA volume index, mL/m^2^, median (IQR)	29 (25–37)	31 (24–36)	29 (25–37)	0.09

Abbreviations: ECG = electrocardiogram; dur. = duration; neg. = negative; amp. = amplitude; LA = left atrial; plax = parasternal long-axis view; ap4 = apical 4-chamber view; ap2 = apical 2-chamber view; IQR = interquartile range; n = number.

**Table 3:** Multivariate Analysis.

Characteristics	Multivariate *p*-Value	Exp (β)
**Baseline characteristics**		
Age	0.01	1.09
Sex	0.48	1.56
CHA_2_DS_2_-Vasc Score	0.34	0.75
Congestive heart failure	0.33	4.30
**ECG parameters**		
Premature atrial contractions present, n (%)	<0.001	27.83
Echocardiographic parameters		
LA volume index	0.74	1.01

Abbreviations: ECG = electrocardiogram; LA = left atrial; n = number.

## Data Availability

The data presented in this study are available on request from the corresponding author. The date are not privately available due to privacy restrictions.
